# A Data Reconstruction Method for Inspection Mode in GBSAR Monitoring Using Sage–Husa Adaptive Kalman Filtering and RTS Smoothing

**DOI:** 10.3390/s25133937

**Published:** 2025-06-24

**Authors:** Yaolong Qi, Jialiang Guo, Jiaxin Hui, Ting Hou, Pingping Huang, Weixian Tan, Wei Xu

**Affiliations:** 1 College of Information Engineering, Inner Mongolia University of Technology, Hohhot 010051, China; qiyaolong@imut.edu.cn (Y.Q.); 15352876642@163.com (J.G.); h2353854694@163.com (J.H.); hwangpp@imut.edu.cn (P.H.); wxtan@imut.edu.cn (W.T.); xuwei1983@imut.edu.cn (W.X.); 2 Inner Mongolia Key Laboratory of Radar Technology and Application, Hohhot 010051, China

**Keywords:** GB-SAR, RTS smoothing, inspection mode, Sage–Husa Kalman filter, data reconstruction

## Abstract

Ground-based synthetic aperture radar (GBSAR) has been widely used in the fields of early warning of geologic hazards and deformation monitoring of engineering structures due to its characteristics of high spatial resolution, zero spatial baseline, and short revisit period. However, in the continuous monitoring process of GBSAR, due to the sudden failure of radar equipment, such as power failure, or the influence of alternating work between multiple regions, it often leads to discontinuous data collection, and this problem caused by missing data is collectively called “inspection mode”. The problem of missing data in the inspection mode not only destroys the spatial and temporal continuity of the data but also affects the accuracy of the subsequent deformation analysis. In order to solve this problem, in this paper, we propose a data reconstruction method that combines Sage–Husa Kalman adaptive filtering and the Rauch–Tung–Striebel (RTS) smoothing algorithm. The method is based on the principle of Kalman filtering and solves the problem of “model mismatch” caused by the fixed noise statistics of traditional Kalman filtering by dynamically adjusting the noise covariance to adapt to the non-stationary characteristics of the observed data. Subsequently, the Rauch–Tung–Striebel (RTS) smoothing algorithm is used to process the preliminary filtering results to eliminate the cumulative error during the period of missing data and recover the complete and smooth deformation time series. The experimental and simulation results show that this method successfully restores the spatial and temporal continuity of the inspection data, thus improving the overall accuracy and stability of deformation monitoring.

## 1. Introduction

In recent years, ground-based synthetic aperture radar (GB-SAR) has been an important tool for early warning of geologic hazards and the deformation monitoring of engineering structures due to its advantages of all-day, all-weather, high-resolution, and non-contact monitoring [1]. Capable of continuously acquiring target-area data, GBSAR provides a reliable foundation for disaster risk analysis and assessment. By constructing a continuous interferometric phase sequence, it enables dynamic sensing of sub-millimeter deformation in target areas, providing multi-dimensional deformation information for disaster mechanism analysis and quantitative risk assessment [2,3,4]. However, in the actual engineering monitoring process, data acquisition is often intermittent due to the interference of various factors such as instrument maintenance, power interruption due to extreme weather conditions, and rotational patrol operation at multiple monitoring sites. In this paper, this discontinuous acquisition state is collectively referred to as “inspection mode”. The problem of missing data in the inspection mode not only destroys the spatial and temporal continuity of the data but also leads to the deviation of the subsequent deformation analysis, which in turn affects the overall accuracy and stability of the monitoring system [5].

The Kalman filtering technique, as a powerful dynamic system estimation method, is widely used in all kinds of signal processing fields, especially in the treatment of missing data and noise interference, with significant advantages [6]. Kalman filtering is based on the state–space model, with Zeng Xin Li et al. improving the accuracy of landslide deformation prediction by combining the gray model and Kalman filtering [7].

In terms of algorithmic improvements, Estahbanati A.T. proposed the extended Kalman filter (EKF) method to effectively solve the phase de-entanglement problem [8]. In the field of landslide deformation prediction, scholars have made a series of breakthrough achievements. Acar used the Kalman filter algorithm to carry out a dynamic and static deformation comparison study on the GPS monitoring data of the landslide area and verified the effectiveness of the Kalman filter [9]. In the study by Hastaoğlu, based on the Kalman filter algorithm, the long-term observation data of the landslide area were systematically analyzed. The results showed that the dynamic deformation model was better than the static model in terms of displacement prediction accuracy [10]. And Mosbeh R. Kaloop applied the least squares (PLS) method in combination with Kalman filtering to deformation monitoring, and the effectiveness of the method was proven through comparative experiments [11]. Francesco Lattari et al. significantly improved the performance of SAR image denoising by augmenting multiscale feature extraction with the encoder–decoder structure and hopping connections, combined with a two-phase training strategy (synthetic data pre-training + real data fine-tuning) and adaptive TV regularization [12]. In a high-precision monitoring system used in the study by Wang Z. et al. a real-time ground-based SAR (RT-GBSAR) processing chain based on the small baseline set (SBAS) concept significantly reduced the computational memory requirement through a unitized processing strategy and achieved continuous deformation monitoring with sub-millimeter accuracy [13]. Qiao H. et al. used UAV Ku-band synthetic aperture radar tomography (TomoSAR) to reconstruct a snow layer profile and realized high-precision inversion of the snow layer structure through the comparison of three algorithms, MUSIC, Capon, and Beamforming, and the correction of medium refraction, which provided methodological support for the large-scale monitoring of the snow layer via satellite-mounted SAR [14]. Kalman filtering exhibits a strong fusion ability. Manoochehr Shirzaei utilized Kalman and wavelet filters to efficiently suppress errors caused by orbital viewpoint differences and atmospheric delays in synthetic aperture radar [15]. A GPS real-time monitoring system based on the Kalman filter was established by Ince, and the deformation monitoring and analysis of the surrounding area were carried out. It has been proven that the Kalman filter can help to detect outliers and improve the reliability of deformation analysis [16].

Although Kalman filtering has achieved good results in many fields, traditional Kalman filtering algorithms usually assume that the statistical properties of the noise are fixed, which may lead to the “model mismatch” problem in practical applications, especially in complex monitoring environments. In order to solve this problem, this paper proposes a fusion data reconstruction method based on the Sage–Husa Kalman adaptive filter and the Rauch–Tung–Striebel (RTS) smoothing algorithm, which adaptively adjust the noise covariance matrix according to the dynamics of the data, thus improving the performance and estimation accuracy of the system in non-stationary environments. Meanwhile, the RTS smoothing algorithm further improves restoration accuracy in cases of prolonged data gaps by globally optimizing the forward filtering results and correcting estimation errors in earlier periods using future observations. The experimental results show that the method not only significantly improves the accuracy of deformation monitoring but also enhances the stability of the monitoring system, which provides more reliable technical support for the early warning of geologic hazards and health monitoring of engineering structures. In conclusion, Sage–Husa adaptive filtering adapts to the complex monitoring environment by dynamically adjusting the noise parameters, while the RTS smoothing algorithm further improves reconstruction accuracy under long-time data loss through global optimization, and the synergistic effect of the two ensures the validity and robustness of the method in practical applications.

## 2. Methods

Since the data acquisition in inspection mode is often intermittent, the statistical characteristics of noise change greatly over time, and the fixed noise model can easily lead to the “model mismatch” problem, while Sage–Husa adaptive filtering can improve the accuracy of state estimation by dynamically adjusting the noise covariance matrix; at the same time, RTS backward smoothing can further reduce the accumulation of errors caused by the lack of data over long time periods. RTS backward smoothing utilizes the future observation information to globally optimize the forward filtering results, which further reduces the error accumulation caused by the missing data over long time periods. This section details how Sage–Husa adaptive Kalman filtering and the RTS backward smoothing fusion algorithm are applied to reconstruct the foundation SAR deformation monitoring data.

### 2.1. Sage–Husa Adaptive Kalman Filtering

In the traditional Kalman filtering algorithm, the covariance matrices Q and R of the process noise $\omega_{k}$ and the observation noise $v_{k}$ are usually assumed to be fixed constants, i.e., the system satisfies the following model:

(1) $$x_{k+1}=F_{k}x_{k}+\omega_{k},\omega_{k}∼N\left(0,Q_{k}\right)$$

(2) $$z_{k}=H_{k}x_{k}+v_{k},v_{k}∼N\left(0,R_{k}\right)$$

However, in the actual monitoring process, the statistical properties of the noise often change with time and environment, resulting in a fixed Q and R that cannot accurately describe the uncertainty of the system, thus degrading the filtering performance. For this reason, Sage–Husa adaptive filtering is able to adaptively adjust the noise parameters according to the residuals between prediction and observation during the filtering process by introducing an adaptive factor, which in turn improves state estimation accuracy.

Considering the displacement changes in the observed region, the state vector is defined as follows:

(3) $$X_{k}=\left[\begin{array}{c} p_{k}\\ v_{k}\\ a_{k} \end{array}\right]$$

where $p_{k}$ is the cumulative deformation, $v_{k}$ is the deformation rate, and $a_{k}$ is the acceleration. Based on the motion characteristics of the monitored target, the system state transfer model can be switched adaptively according to the motion of the target. For a faster moving target, the constant acceleration model is used, and its state transfer matrix is as follows:

(4) $$F_{acc}=\left[\begin{array}{ccc} 1 & \Delta t & \frac{1}{2}\Delta t^{2}\\ 0 & 1 & \Delta t\\ 0 & 0 & 1 \end{array}\right]$$

When the target moves slowly or is at rest, in order to minimize the effect of noise on acceleration estimation, a constant velocity model is used with a state transfer matrix:

(5) $$F_{cs}=\left[\begin{array}{ccc} 1 & \Delta t & 0\\ 0 & 1 & 0\\ 0 & 0 & 1 \end{array}\right]$$

The observation equation is then:

(6) $$Z_{k}=HX_{k}+v_{k}$$

(7) $$H=\left[1\;0\;0\right]$$

In the standard Kalman filter prediction step, the recursive formula for state and covariance is as follows:

(8) $$X_{k,k-1}=F_{k}X_{k-1}$$

(9) $$P_{k,k-1}=F_{k}P_{k-1}F_{k}^{T}+Q_{k}$$

In order to balance the influence of historical information and current observation in the filtering process, the Sage–Husa algorithm introduces an adaptive factor $\gamma_{k}$ for dynamic adjustment, which is calculated as follows:

(10) $$\gamma_{k}=\frac{1-b}{1-b^{k}}$$

where b is a forgetting factor, which is mainly used to conduct multiple trials to select the optimal result, and the range of values is determined between 0.95 and 0.99 to control the contribution of historical data to the current estimate. In the measurement update step, the prediction residuals are computed if valid observations currently exist:

(11) $$e_{k}=z_{k}-Hx_{k,k-1}$$

The residual information is then used to correct the observation noise covariance matrix $R_{k}$ so that it can adaptively reflect changes in the observation:

(12) $$R_{k}=\left(1-\gamma_{k}\right)R_{k-1}+\gamma_{k}\left(e_{k}e_{k}^{T}-H_{k}P_{k,k-1}H_{k}^{T}\right)$$

Next, the Kalman gain is calculated using the updated $R_{k}$:

(13) $$K_{k}=P_{k,k-1}H^{T}{\left(HP_{k|k-1}H^{T}+R_{k}\right)}^{-1}$$

And we update the state estimates and covariance matrix:

(14) X^k,k=X^k,k−1+Kkek

(15) $$P_{k,k}=\left(I-K_{k}H_{k}\right)P_{k,k-1}$$

In patrol mode, due to the lack of valid observations at some moments, the filter is unable to compute the residual $e_{k}$ for regular measurement updating, in which case the filter skips the measurement updating and relies only on the prediction step for state recursion. To compensate for the uncertainty caused by the lack of observation information, this paper utilizes the prediction covariance matrix to reflect the uncertainty in the propagation process of the system and adaptively adjusts the process noise covariance $Q_{k}$ based on it:

(16) $$Q_{k}=\left(1-\gamma_{k}\right)Q_{k-1}+\gamma_{k}P_{k,k}$$

In this way, even during periods of missing data, the filter is able to rationalize the process noise to the uncertainty of the system, thus reducing the accumulation of prediction errors. Once valid observations are restored, the filter is able to quickly correct the state and converge to an accurate estimate using the measurement update formula.

### 2.2. RTS Model

Although Sage–Husa adaptive filtering can effectively update the noise parameters during the recursion process and improve state estimation accuracy, the cumulative error of the forward recursion may be larger in the case of missing data due to the limitation of forward filtering with gradual error transfer. In order to fully utilize the global information and further correct the forward filtering results, the RTS backward smoothing algorithm is introduced into this method. The RTS smoothing algorithm is a fixed-interval smoother based on the great likelihood estimation criterion derived from the forward and backward filtering by Rauth et al. It has low computational requirements and is an efficient and optimal smoothing method, with it being widely used in various fields [17].

In the Sage–Husa adaptive Kalman filter, the state estimate X^k,k and its covariance $P_{k,k}$ are obtained at each moment after the measurement update, and they are denoted as X^kf and $P_{k}^{f}$, respectively, in order to discriminate them from the posterior filters. at the same time, the state estimate X^kf is predicted to be the state estimate for the “k+1 ”th moment according to the state transfer model X^k+1,k and the predicted covariance $P_{k+1,k}$ The basic idea of RTS smoothing is to utilize the smoothing results of the subsequent moments to correct the forward filtering results, so as to make the entire state sequence fully integrate the information of the past and the future and achieve global optimization.

The core of the RTS smoothing process is the calculation of the smoothing gain, which is given by the following:

(17) $$G_{k}=P_{k}^{f}F_{k}^{T}{\left(P_{k+1,k}\right)}^{-1}$$

where $F_{k}$ is the state transfer matrix and $G_{k}$ is the smoothing gain, $G_{k}$ can feed the smoothing information of the “k + 1” moment into the filtering result of the moment k, so as to correct the error due to local noise and missing data.

The smooth update formula for the state is as follows:

(18) X^k,k=X^k,k−1+Kkek

The smoothing correction formula for the covariance is as follows:

(19) $$P_{k}^{s}=P_{k}^{f}+G_{k}\left(P_{k+1}^{s}-P_{k+1,k}\right)G_{k}^{T}$$

where X^ks and $P_{k}^{s}$ denote the state estimate and its uncertainty at the kth moment after RTS smoothing, respectively.

RTS smoothing usually starts at the end of the observation sequence, setting the smoothing result of the last moment as the forward filtering result, i.e., making X^Ns=X^kf and $P_{N}^{s}=P_{N}^{f}$ and then sequentially recursively moving forward to correct the states of the previous moments using the subsequent data. This requires complete preservation of the state estimates, filtered covariances, and the sequence of states and predicted covariances obtained based on the state-shift prediction during the forward filtering process. Using these historical data, the global information can be fully integrated into the backward recursion, and the local deviations due to noise changes or data discontinuities in the filtering process can be fully corrected, so as to realize a more accurate and smoother state reconstruction.

**Figure 1:**
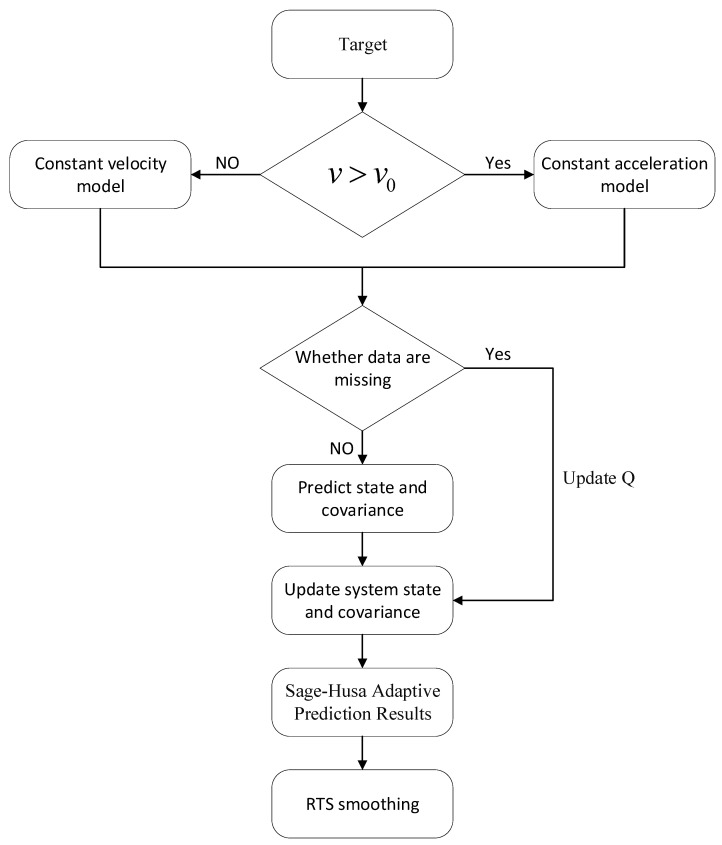
Algorithm flowchart.

By combining the Sage–Husa adaptive Kalman filter with the RTS backward smoothing algorithm, the problem of missing data in the ground-based SAR inspection mode can be effectively solved. The introduction of the adaptive factor enables the observation noise covariance to be dynamically adjusted with environmental changes and instrument fluctuations, which not only avoids the influence of unstable noise statistics on state estimation and makes the estimation results more in line with the actual situation but also effectively reconstructs the missing data and improves the completeness of the deformation time series in the inspection mode. RTS backward smoothing relies on the inverse recursion, which integrates the observation information of the future moments into the calculation process and provides a better solution to the problem of forward filtering. RTS backward smoothing relies on backward recursion to incorporate future observation information into the computation process, which globally corrects the error of forward filtering and significantly reduces the impact of error accumulation. The flow chart of the algorithm is shown in Figure 1.

For all missing frame intervals, if the deformation rate $v_{k}$ exceeds the preset threshold $v_{0}$ (i.e., $V>V_{0}$), the constant acceleration model (state transition matrix $F_{acc}$) is adopted to capture the nonlinear dynamics of rapidly deforming targets such as landslide areas. Otherwise, the system switches to the constant velocity model ($F_{cs}$) to suppress noise interference in stable regions. The core loop dynamically executes Sage–Husa adaptive filtering based on data availability: when observations exist, it utilizes residuals to update the observation noise covariance $R_{k}$ online and corrects the state; during data gaps, it adaptively adjusts the process noise covariance $Q_{k}$ via the prediction covariance (the “Update Q” node), effectively curbing error accumulation. Finally, through the RTS backward smoothing module, information from the entire time series is fused recursively to correct local errors from forward filtering using the smoothing gain $G_{k}$, outputting a continuous deformation sequence.

## 3. Experimental Results

### 3.1. Study Area and Equipment

**Figure 2:**
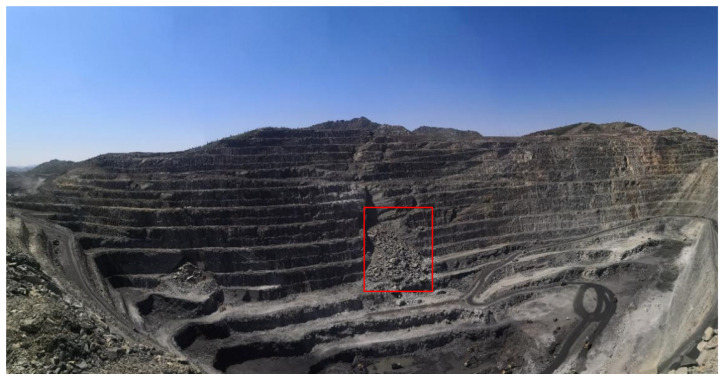
Optical image of the study area in situ.

An open-pit mine in northwestern China was selected as the study area for this experiment, and 217 SAR images were chosen to cover the time from 0:00 a.m. on 18 April 2021 to 10:00 a.m. on 21 April 2021. It is located in the plateau area with an elevation of 1000–1300 m, showing typical north-high–east-low topographic features, with a relative elevation difference of 50–300 m in the area. The geological structure of the mine is mainly composed of rocks. The experiment adopts a slope-oriented radar deployment scheme, which realizes effective coverage of the entire mining area. The optical image of the site is shown in Figure 2.

**Figure 3:**
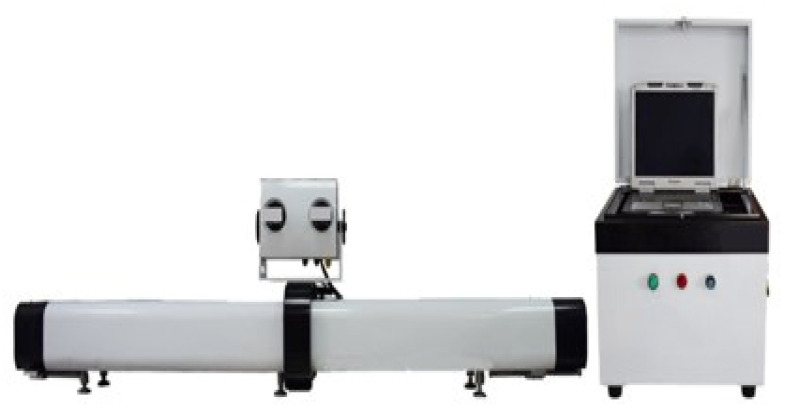
GB-SAR system.

The red boxes in the figure mark the locations where landslides occurred during the monitoring period. The instrument used for the observations was a line-scanning microdeformation monitoring radar, a Ku-band GB-InSAR system. The radar picture is shown in Figure 3, and the system parameters are shown in Table 1.

### 3.2. Experimental Results and Analysis

#### 3.2.1. Missing Data at Different Monitoring Points

In this experiment, we processed data derived from 217 interferograms, utilizing atmospheric phase correction and deformation inversion techniques to generate a cumulative deformation map. To accommodate practical monitoring requirements, a velocity threshold was implemented to enable adaptive switching between state models. Specifically, a constant acceleration model was applied in landslide-prone regions characterized by rapid deformation rates, while stable regions employed a constant velocity model to more effectively mitigate noise interference.

During periods of missing data, filters were recursively derived based on state predictions, and an adaptive update mechanism, guided by prediction covariance, dynamically adjusted the process noise parameter (Q). This strategy allowed for the estimation of system uncertainties during data gaps and facilitated rapid correction upon the restoration of valid observational data.

**Figure 4:**
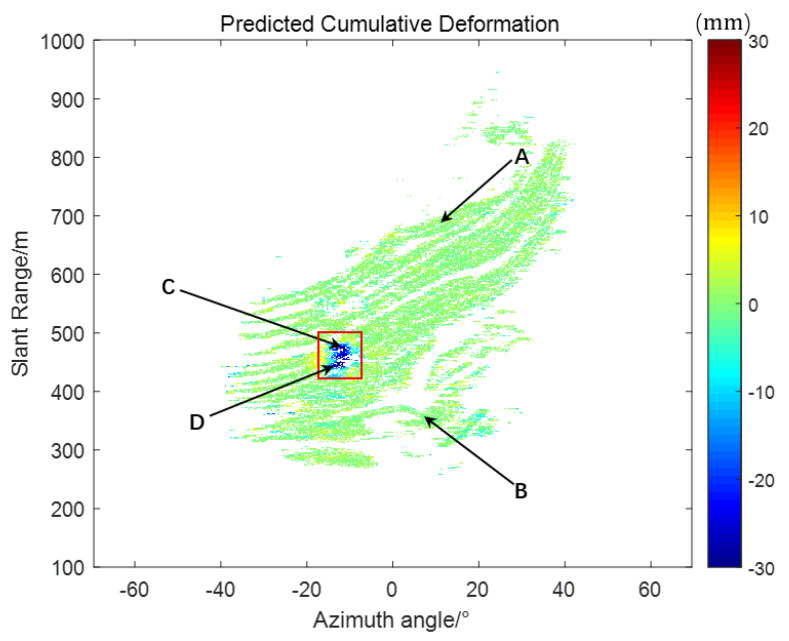
Cumulative deformation area.

**Figure 5:**
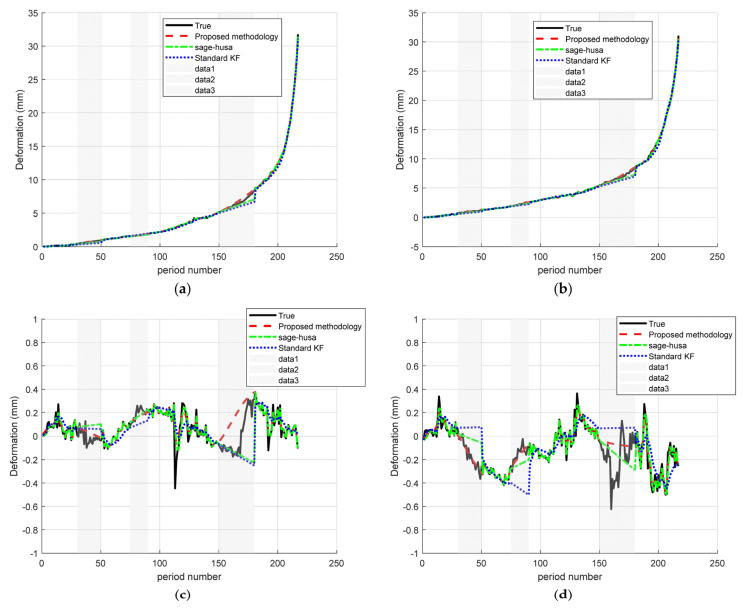
Time–series deformation curves for monitoring sites: (**a**) monitoring site A; (**b**) monitoring site B; (**c**) monitoring site C; (**d**) monitoring site D.

To evaluate the robustness of the proposed fusion algorithm under varying data-loss scenarios, four representative monitoring points were selected—two from dynamic deformation zones and two from stable regions. Points A (−11.61, 472.5) and B (−12.83, 443.1) demonstrated significant deformation activity, whereas points C (10.69, 686.4) and D (−22.61, 551.1) exhibited minimal or no movement, remaining effectively stationary (Figure 4). The results are shown in Figure 5.

Table 2 shows that the proposed fusion algorithm achieves the lowest MAE and RMSE at all monitoring points, and at monitoring point A, the MAE/RMSE of the standard Kalman filter is 0.195/0.333, which is reduced to 0.083/0.204 for Sage–Husa, while that for the improved algorithm is only 0.057/0.125, compared with the standard KF by about 71% and 62%, respectively. At Site B, the standard KF is reduced by about 71% and 62% compared to the standard KF, and further improved by about 31% and 39% compared to the Sage–Husa. At monitoring point B, the MAE/RMSE of the standard KF is 0.203/0.332, and that of Sage–Husa is 0.093/0.198, while that of the improved algorithm is reduced to 0.051/0.100, which is reduced by about 75%/70% compared to the standard KF, and 45% 45% compared to Sage–Husa, respectively. Compared with the standard KF, it is reduced by about 75%/70%, and compared with Sage–Husa, it is reduced by about 45%/49%. At monitoring point C, the errors are reduced from 0.080/0.132 for standard KF and 0.048/0.114 for Sage–Husa to 0.045/0.090 for the improved algorithm, and at monitoring point D, the errors are reduced from 0.127/0.180 for standard KF and 0.054/0.099 for Sage–Husa to 0.045/0.090 for the improved algorithm.

Overall, the Sage–Husa + RTS fusion method consistently achieves the lowest error metrics at each site, with a 25–45% reduction in MAE (20–40% reduction in RMSE) compared to Sage–Husa, and a 54–75% reduction in MAE (62–70% reduction in RMSE) compared to standard KF. In addition, the error magnitude decreases gradually from point A to point D, reflecting the enhanced stability in the low-interference region. These results confirm the high accuracy and robustness of the proposed fusion algorithm in different deformation environments.

#### 3.2.2. Missing Data in Multiple Scenarios

In order to deeply evaluate the performance of the proposed fusion algorithm in real data loss situations, we designed three typical data loss patterns and tested them at two key measurement points (point A: −11.61, 472.5; point D: −22.61, 551.1). The missing data scenarios were (1) sudden large data loss mixed with scattered small missing segments, (2) continuous missing data accompanied by phase changes, and (3) multiple intermittent missing data of moderate length. The reconstruction accuracy is quantified by utilizing the mean absolute error (MAE) and root mean square error (RMSE).

**Figure 6:**
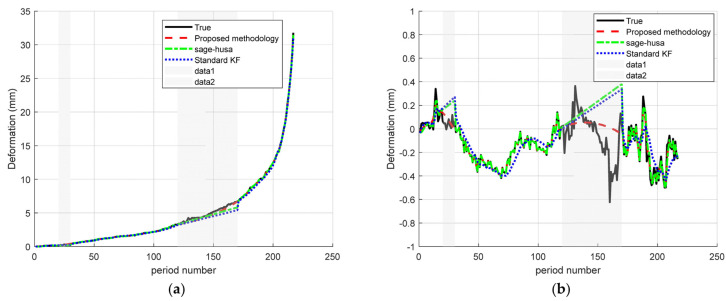
Time–series deformation curves with large missing segments plus small randomly missing segments: (**a**) monitoring site A; (**b**) monitoring site D.

In the scenario of “Segments plus random small missing segments (omit ranges = [20–30; 120–170])”, a number of small missing segments are inserted into the entire sequence to reproduce extreme packet loss or equipment failures that may occur during the observation. The results are shown in the Figure 6.

In the scenario of “Missing large segments plus randomized missing small segments” (omit ranges = [20–30; 120–170]), the Sage–Husa + RTS fusion algorithm proposed in this paper achieves significant reconstruction accuracy improvements at both points A and D. Both points A and D achieve significant reconstruction accuracy improvement. As shown in Figure 6 and the results in Table 3, compared with the standard Kalman filter, the MAE at point A decreases from 0.223 to 0.074 (down by about 66.7%) and the RMSE decreases from 0.374 to 0.144 (down by about 61.5%), while at point D, the MAE decreases from 0.119 to 0.047 (down by about 60.5%) and the RMSE decreases from 0.189 to 0.101 (a decrease of about 46.6%). Compared with the Sage–Husa algorithm, the MAE/RMSE are additionally reduced by about 25.3%/35.4% at point A and 42.7%/44.8% at point D, respectively. Our experimental results show that the proposed fusion algorithm achieves smooth, oscillation-free, and high-precision signal reconstruction in the presence of severe missing data.

**Figure 7:**
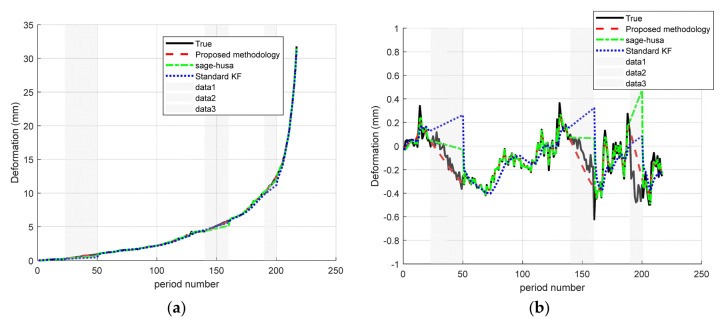
Time–series deformation curves for early, intermediate, and end-stage data lacking predictive power: (**a**) monitoring site A and (**b**) monitoring site D.

In the second scenario, there are consecutive loses in the “early (23–50), middle (140–160), and late (190–200) middle (140–160), and late (190–200)”, respectively. The results are shown in the Figure 7.

Under the scenarios of “early (23–50), middle (140–160), and late (190–200) consecutive loss”, the proposed Sage–Husa + RTS fusion algorithm shows excellent prediction ability for both A and D points. Specifically, as shown in Figure 7 and Table 4, the MAE/RMSE at point A is reduced from 0.162/0.261 in the standard KF to 0.031/0.064, which is about 80.8% and 75.5% lower than that in the standard KF, and an additional 60.8%/62.6% lower than that in Sage–Husa (0.079/0.171). 62.6%; the MAE/RMSE at point D decreased from 0.119/0.189 to 0.047/0.101, which was about 60.5%/46.6% lower than that of the standard KF, and 42.7%/44.8% lower than that of Sage–Husa (0.082/0.183). The above results show that the present method can realize high-precision, smooth, and oscillation-free interpolation reconstruction when large consecutive missing segments occur at any stage of the sequence, which fully verifies its robust prediction ability for early, intermediate, and final missing data.

**Figure 8:**
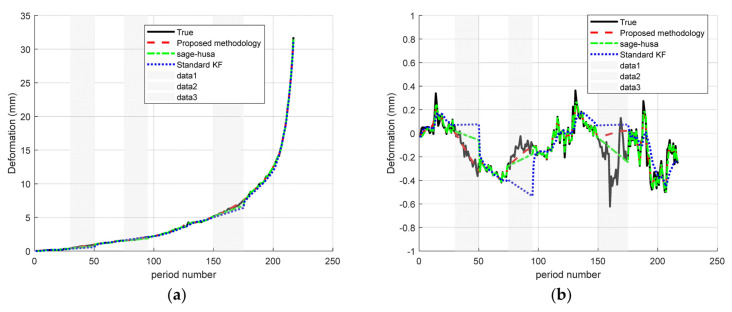
Time–series deformation curves for multiple medium-duration missing data: (**a**) monitoring site A and (**b**) monitoring site D.

The third scenario is “multiple medium-length missing”. The results are shown in the figure below (Figure 8).

In the “multiple medium-length missing” scenario (omit_ranges = [30–50; 75–95; 150–175]), as shown in Table 5: the proposed Sage–Husa + RTS fusion algorithm delivers a MAE/RMSE of 0.032/0.061 at Point A and 0.046/0.098 at Point B, markedly outperforming both the standard KF (0.170/0.253 and 0.134/0.190) and Sage–Husa alone (0.062/0.127 and 0.050/0.092). Relative to the standard KF, the MAE/RMSE are reduced by approximately 81%/76% at Point A and 66%/48% at Point B; compared with Sage–Husa, the MAE falls by 48%/8% and the RMSE falls by 52%/+6% at A/B, respectively. These results confirm that the proposed fusion method maintains smooth, oscillation-free, high-precision signal reconstruction even when faced with multiple intermittent gaps of moderate duration.

In conclusion, the proposed Sage–Husa + RTS fusion algorithm shows excellent reconstruction capabilities in three typical loss scenarios: (1) large burst loss interspersed with scattered small segment loss, (2) continuous loss in early/mid/late sequence, and (3) multiple intermittent loss of moderate duration—all show excellent reconstruction ability. The method consistently achieves the lowest MAE and RMSE at key measurement points such as A and D: an additional reduction in MAE of ~61–81% and RMSE of ~46–76% compared to the standard KF and a further reduction in MAE of ~25–49% and RMSE of ~1–76% compared to the Sage–Husa single-algorithm. Compared with the Sage–Husa single algorithm, it can further reduce the MAE by about 25–49% and the RMSE by about 35–62%. More importantly, the fusion reconstruction curves remain smooth and oscillation-free in all missing intervals, and there is no over-smoothing phenomenon, which fully proves that the algorithm has high accuracy and strong robustness in both high- and low-interference environments.

#### 3.2.3. Global Cumulative Deformation Analysis

**Figure 9:**
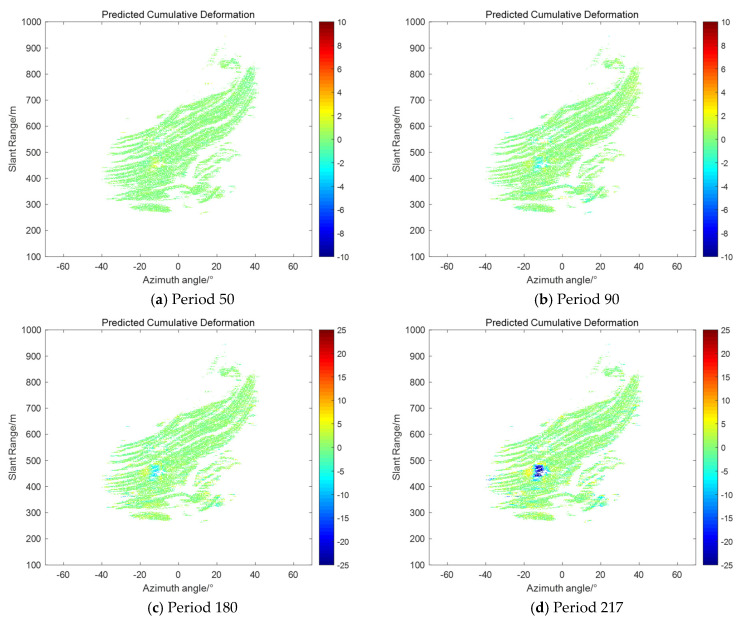
Predicted cumulative deformation map.

Based on the comparative analysis of individual monitoring sites, we further conducted a global validation of the entire monitoring region in order to comprehensively assess the applicability and performance of the proposed method in different regions and deformation characteristics. As shown in Figure 9, we selected the predicted cumulative deformation maps for periods of 50, 90, 180, and 217 for analysis. The first three data periods are at the stage where the observed data are just recovered and can be used to test the recovery effect after a long period of missing data. The 217th period, on the other hand, as the last output period of the entire prediction cycle, can fully reflect the ability of the filtering method to correct the system state during the entire cycle. Specifically, Figure 9a demonstrates the predicted cumulative deformation map for the 50th period, and the maximum deformation in the landslide region is about 2 mm, at which time the trend of deformation is not yet clearly visible. Figure 9b shows the predicted cumulative deformation for period 90, where the maximum deformation in the landslide area increases to about 3.5 mm, but the change is still relatively small. Figure 9c shows the predicted cumulative deformation map for period 180, where the maximum deformation in the landslide area has reached 6 mm and the deformation trend becomes more pronounced. Finally, Figure 9d shows the predicted cumulative deformation map for period 217, where the maximum deformation of the landslide area has exceeded 30 mm, and landslides have already occurred at the site, and the overall deformation trend is consistent with the deformation curve in Figure 9b. Through these analyses, it can be seen that the proposed method can effectively predict and correct deformation changes at different stages, especially during data recovery and long-term prediction, and can accurately reflect the deformation characteristics of the landslide area.

Table 6 demonstrates the root mean square error (RMSE) of the proposed fusion algorithm versus the Sage–Husa adaptive Kalman filtering method alone in predicting cumulative deformation. From the statistical results, the RMSE values are significantly larger when using the Sage–Husa adaptive Kalman filter alone after the 180th prediction period, reflecting the failure of the state model to accurately characterize the system’s changes at this point in time, which leads to a more serious accumulation of errors. Although the Kalman filtering method was able to make adjustments quickly after the observed data were recovered, and the RMSE improved by the 217th period, its overall error level was still higher than that of the fusion method using the RTS backward smoothing algorithm. This result suggests that although the Kalman filter method is able to make rapid corrections after data recovery, the accumulation of errors is still more pronounced during the long-term prediction process, especially during periods of missing data. The fusion algorithm, however, can more effectively reduce the number of errors and improve the accuracy of deformation prediction by combining with RTS backward smoothing.

## 4. Summary and Discussion

In this paper, we propose a data reconstruction method that combines Sage–Husa adaptive Kalman filtering and the Rauch–Tung–Striebel (RTS) smoothing algorithm to address the problem of missing data in the inspection mode of the ground-based synthetic aperture radar (GBSAR) monitoring process due to the maintenance of the equipment and the rotational patrol operation. Data reconstruction method: By introducing an adaptive forgetting factor, the method dynamically adjusts the covariance matrix between the process noise and the observation noise, which effectively solves the problem of “model mismatch” caused by the fixed statistical characteristics of the noise in the traditional Kalman filtering algorithm. In addition, the RTS smoothing algorithm globally optimizes the forward filtering results through backward recursion, which significantly reduces the error accumulation in the long missing data period.

Through experimental validation at multiple key monitoring points in real mining areas, the proposed algorithm shows excellent reconstruction results in different deformation feature regions and multiple typical missing data scenarios (including continuous missing, random missing, and multiple medium-length missing segments). The results show that the mean absolute error (MAE) and root mean square error (RMSE) of the method are reduced by about 54–81% and 46–76%, respectively, compared with the standard Kalman filtering algorithm. In practical applications, the method effectively improves the spatial continuity and temporal stability of the data, demonstrates high accuracy and no excessive smoothing, and can be widely used in the fields of geologic disaster monitoring and early warning and engineering structure health monitoring.

It is worth noting that there are still some limitations to this method. Especially in the case of more intense deformation velocity and longer observation interval, the selection of process model and noise model has a significant influence on the filtering results, so the switching conditions of constant velocity and constant acceleration models, the forgetting factor, and the filtering gain strategy need to be more carefully adjusted according to the actual application scenarios. In addition, outside of the inspection mode, if the observation data themselves have large fluctuations (such as clutter interference or serious influence from the external environment), they still need to be combined with atmospheric correction and scattering characteristic evaluation.

Future research can be considered in the following directions:

1. The multi-target and multi-sensor fusion technique builds a cross-scale, multi-modal collaborative monitoring system by integrating heterogeneous sensor data such as GBSAR, GPS, and terrestrial laser scanning. The fusion framework employs joint filtering algorithms to take full advantage of the complementary strengths of the different sensors: GBSAR provides high-precision micro-deformation monitoring, GPS ensures an absolute positioning datum, and laser scanning contributes high-resolution three-dimensional spatial information. Through spatial and temporal alignment and error compensation mechanisms, the system can effectively suppress the limitations of a single sensor and significantly improve the reliability of the monitoring data and the adaptability of the algorithm under complex environmental conditions.
2. The reason for the slow processing speed of the current algorithm is that it needs to acquire the complete forward propagation data and then carry out backward smoothing optimization as a whole, resulting in poor real-time performance. In future research, we can consider further optimizing the algorithm architecture, such as introducing a GPU parallel computing mechanism to accelerate the forward data acquisition and backward smoothing operation process, so as to significantly improve the overall operational efficiency of the algorithm and better meet the real-time and performance requirements of practical application scenarios.

Overall, the organic combination of Sage–Husa adaptive filtering and RTS smoothing can effectively deal with the problem of incomplete deformation sequences caused by the interruption of data acquisition in the foundation SAR inspection mode and improve the accuracy and smoothness of monitoring data. The experimental results verify the effectiveness and universality of the method in different regions (including stabilized and landslide zones) and provide a new idea for the applications of the early warning of geohazards and deformation monitoring of engineering structures.

## Figures and Tables

**Table 1:** Radar system parameters.

Parameter	Value
Distance resolution	0.2 m
Azimuth resolution	0.3°
Band	Ku
Sampling interval	20 min
Bandwidth	500 MHz
Working distance	10 m to 5 km
Visual angle	>60°(azi.) × 30°(ele.)
Revisit period	8 min

**Table 2:** Comparison of the MAE and RMSE of the different filtering methods at each monitoring point.

Monitoring Point	Sage–Husa + RTS	Sage–Husa	Standard KF
Point A	MAE = 0.057	MAE = 0.083	MAE = 0.195
	RMSE = 0.125	RMSE = 0.204	RMSE = 0.333
Point B	MAE = 0.051	MAE = 0.093	MAE = 0.203
	RMSE = 0.100	RMSE = 0.198	RMSE = 0.332
Point C	MAE = 0.045	MAE = 0.048	MAE = 0.080
	RMSE = 0.090	RMSE = 0.114	RMSE = 0.132
Point D	MAE = 0.041	MAE = 0.054	MAE = 0.127
	RMSE = 0.082	RMSE = 0.099	RMSE = 0.180

**Table 3:** Comparison of the MAE and RMSE of the different filtering methods for missing large segments plus randomized missing small segments.

Monitoring Point	Sage–Husa + RTS	Sage–Husa	Standard KF
Point A	MAE = 0.074	MAE = 0.099	MAE = 0.223
	RMSE = 0.144	RMSE = 0.223	RMSE = 0.374
Point D	MAE = 0.047	MAE = 0.082	MAE = 0.119
	RMSE = 0.101	RMSE = 0.183	RMSE = 0.189

**Table 4:** Comparison of the MAE and RMSE of early, intermediate, and final data.

Monitoring Point	Sage–Husa + RTS	Sage–Husa	Standard KF
Point A	MAE = 0.031	MAE = 0.079	MAE = 0.162
	RMSE = 0.064	RMSE = 0.171	RMSE = 0.261
Point D	MAE = 0.047	MAE = 0.082	MAE = 0.119
	RMSE = 0.101	RMSE = 0.183	RMSE = 0.189

**Table 5:** Comparison of the MAE and RMSE of multiple medium-duration missing data.

Monitoring Point	Sage–Husa + RTS	Sage–Husa	Standard KF
Point A	MAE = 0.032	MAE = 0.062	MAE = 0.170
	RMSE = 0.061	RMSE = 0.127	RMSE = 0.253
Point D	MAE = 0.046	MAE = 0.050	MAE = 0.134
	RMSE = 0.098	RMSE = 0.092	RMSE = 0.190

**Table 6:** Root mean square error of deformation prediction.

Methods	50th	90th	180th	217th
Sage–Husa	0.0874	0.0723	0.3436	0.095
Sage–Husa + RTS	0.0212	0.02	0.0653	0.0631

## Data Availability

Data are contained within the article.
